# Uteroplacental nutrient flux and evidence for metabolic reprogramming during sustained hypoxemia

**DOI:** 10.14814/phy2.15033

**Published:** 2021-09-23

**Authors:** Amanda K. Jones, Paul J. Rozance, Laura D. Brown, Ramón A. Lorca, Colleen G. Julian, Lorna G. Moore, Sean W. Limesand, Stephanie R. Wesolowski

**Affiliations:** ^1^ Perinatal Research Center, Department of Pediatrics University of Colorado School of Medicine Aurora Colorado USA; ^2^ Department of Obstetrics and Gynecology University of Colorado School of Medicine Aurora Colorado USA; ^3^ Department of Medicine University of Colorado School of Medicine Aurora Colorado USA; ^4^ School of Animal and Comparative Biomedical Sciences University of Arizona Tucson Arizona USA

**Keywords:** fetal, hypoxemia, metabolism, uteroplacental

## Abstract

Gestational hypoxemia is often associated with reduced birth weight, yet how hypoxemia controls uteroplacental nutrient metabolism and supply to the fetus is unclear. This study tested the effects of maternal hypoxemia (HOX) between 0.8 and 0.9 gestation on uteroplacental nutrient metabolism and flux to the fetus in pregnant sheep. Despite hypoxemia, uteroplacental and fetal oxygen utilization and net glucose and lactate uptake rates were similar in HOX (*n *= 11) compared to CON (*n* = 7) groups. HOX fetuses had increased lactate and pyruvate concentrations and increased net pyruvate output to the utero‐placenta. In the HOX group, uteroplacental flux of alanine to the fetus was decreased, as was glutamate flux from the fetus. HOX fetuses had increased alanine and decreased aspartate, serine, and glutamate concentrations. In HOX placental tissue, we identified hypoxic responses that should increase mitochondrial efficiency (decreased *SDHB*, increased *COX4I2*) and increase lactate production from pyruvate (increased LDHA protein and LDH activity, decreased *LDHB* and *MPC2*), both resembling metabolic reprogramming, but with evidence for decreased (*PFK1*, *PKM2*), rather than increased, glycolysis and AMPK phosphorylation. This supports a fetal‐uteroplacental shuttle during sustained hypoxemia whereby uteroplacental tissues produce lactate as fuel for the fetus using pyruvate released from the fetus, rather than pyruvate produced from glucose in the placenta, given the absence of increased uteroplacental glucose uptake and glycolytic gene activation. Together, these results provide new mechanisms for how hypoxemia, independent of AMPK activation, regulates uteroplacental metabolism and nutrient allocation to the fetus, which allow the fetus to defend its oxidative metabolism and growth.

## Graphical abstract

- Sustained late gestation hypoxemia induces metabolic adaptations in the placenta and the allocation of nutrients to the fetus.
- Our data support a fetal‐uteroplacental shuttle whereby uteroplacental tissues produce lactate as fuel for the fetus using pyruvate released from the fetus, rather than pyruvate produced from glucose in the placenta, given the absence of increased uteroplacental glucose uptake and glycolytic gene activation.
- These results provide new mechanisms for how hypoxemia, independent of AMPK activation and reduced blood flow, regulates uteroplacental metabolism and nutrient allocation to the fetus, which allow the fetus to defend its oxidative metabolism and growth.

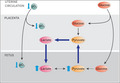

## 1 INTRODUCTION

Fetal hypoxemia is a common feature of pregnancies at high altitude and those affected by ischemic placental disease which includes preeclampsia, chronic placental vascular anomalies, and placental insufficiency‐induced intrauterine growth restriction (IUGR) (Ananth, 2014; Ananth & Vintzileos, 2008; Giussani, 2016). This is important because fetuses exposed to hypoxemia across gestation are often born smaller and have an increased risk for developing more severe intrauterine growth restriction (IUGR) (Ducsay et al., 2018; Giussani et al., 2001; Julian et al., 2008; Keyes et al., 2003; Lackman et al., 2001; Moore, 2021; Moore et al., 2011; Soria et al., 2013; Vaughan et al., 2020). Hypoxemia may limit fetal growth because of changes in placental nutrient metabolism or transport capacity to the fetus. Glucose, lactate, and amino acids are the major substrates for oxidative metabolism and growth in the fetus. Glucose supplied from the mother is transported to the fetus across the placenta by facilitated diffusion (Hay, 1991a; Illsley & Baumann, 2020; Marconi et al., 1996; Vaughan & Fowden, 2016). Lactate is produced endogenously by the fetus and supplied by uteroplacental production (Sparks et al., 1982; Vaughan & Fowden, 2016). Essential amino acids are transported from the maternal to fetal circulation, while nonessential amino acids can be transported or made by the fetus or placenta (Brown et al., 2017; Cetin, 2001; Chung et al., 1998). Importantly, the mechanisms underlying the effects of hypoxemia on the placental allocation of these nutrients to the fetus and how the fetus utilizes the available substrates for oxidative metabolism and growth remain largely unknown.

Metabolic responses to low oxygen availability may play a role mediating the effects of hypoxemia on placental oxidative metabolism during high‐altitude pregnancies (Illsley et al., 2010). Cells respond to decreases in oxygen through hypoxia inducible transcription factors (HIFs) (Semenza, 2001, 2007) which control metabolism by regulating gene expression (Aragones et al., 2009; Rankin et al., 2007). Classic HIF responses include the activation of gene expression for glucose transporters (*GLUT1*), phosphofructokinase (*PFK1*) which controls glycolysis, pyruvate dehydrogenase (PDH) kinases (*PDK1*, *PDK2*, *PDK4*) which inhibit PDH activity and reduce pyruvate (glucose) oxidation, and lactate dehydrogenase A (*LDHA*) which increases lactate production. Other hypoxia transcriptional responses associated with increased glucose utilization and maintenance of oxidation metabolism during hypoxia include increased expression of the pyruvate kinase M2 isoform (*PKM2* vs. *PKM1*), increased cytochrome c oxidase subunit 4 isoform 2 (*COX4I2* vs. *COX4I1*), and decreased succinate dehydrogenase B (*SDHB*). Collectively, many of these responses are known as metabolic reprogramming, a term used to describe how cells adapt their substrate metabolism when oxygen availability is low (DeBerardinis et al., 2008; Illsley et al., 2010). Studies in human pregnancies at high altitude provide some evidence for metabolic reprogramming and have proposed that the placenta increases glucose consumption (glycolysis) without a concomitant increase in oxygen consumption resulting in lactate production, thereby reducing glucose and increasing lactate supply to the fetus (Zamudio et al., 2010). In placental tissue from human high‐altitude pregnancies, however, protein expression of the glucose transporters, GLUT1, and GLUT4, is maintained or decreased (Vaughan et al., 2020; Zamudio et al., 2010), which suggests that any increase in placental glucose consumption occurs without increased glucose transport. Further, acute hypoxia in pregnant sheep did not increase placental uptake of glucose (Tchirikov et al., 2011). Increased glucose utilization in maternal tissues in response to hypoxia also may limit glucose availability to the fetus (Maatta et al., 2018). Thus, hypoxemia may affect uteroplacental glucose and lactate flux to the fetus, yet the molecular pathways involved remain to be fully understood. Further, studies measuring uteroplacental nutrient flux in response to chronic hypoxemia are limited and difficult to perform in humans due to the invasive nature of sampling.

Decreased blood flow to the placenta during hypoxemia may be an additional mechanism that limits umbilical nutrient supply to the fetus. Indeed, uterine and umbilical blood flow is decreased in human pregnancies at high altitude (Julian et al., 2008; Moore, 2021; Zamudio et al., 1995) and during acute experimental hypoxemia (1 h) in pregnant sheep (Tchirikov et al., 2011). Recent studies in murine models of hypoxemia demonstrate that pharmacologic activation of adenosine monophosphate kinase (AMPK) reduces the magnitude of hypoxia‐associated growth restriction with a concomitant rise in uterine artery blood flow (Lane, Doyle, et al., 2020; Lane, Houck, et al., 2020). This supports a mechanistic role for AMPK activation which also may mediate metabolic effects in the placenta, given the role of AMPK as a major nutrient sensor that is activated in response to hypoxia and functions to restore energy balance (Day et al., 2017; Garcia & Shaw, 2017). While decreased placental amino acid transport to the fetus can limit fetal growth (Brown, Regnault, et al., 2017; Cetin, 2001; Vaughan & Fowden, 2016), studies measuring uteroplacental amino acid flux and fetal amino acid concentrations during hypoxemia are limited. A recent analysis reported no differences in amino acid transport capacity in placental tissue from human high‐altitude pregnancies (Vaughan et al., 2020). In addition, mTOR is a major nutrient sensor that coordinates the metabolism of amino acids and other substrates in the placenta (Gupta & Jansson, 2019), yet little is known about its role in uteroplacental nutrient flux during hypoxemia. Thus, hypoxemia may decrease uteroplacental nutrient flux to the fetus via decreased blood flow or placental nutrient sensing and metabolism (Higgins et al., 2016; Illsley et al., 2010; Milley, 1988; Vaughan & Fowden, 2016).

The objective of this study was to determine the effects of sustained hypoxemia on uteroplacental nutrient flux to the fetus. In pregnant sheep, we lowered maternal oxygenation and subsequently reduced fetal oxygenation producing sustained (9 days) hypoxemia in late gestation (Jones, Rozance, et al., 2019). One advantage of the sheep model is the ability to measure uteroplacental oxygen and nutrient transport capacity and test the effects of hypoxemia on uteroplacental nutrient flux to the fetus. In this model, hypoxemic (HOX) fetuses have normal weight‐specific oxygen consumption rates despite a 20% reduction in arterial partial pressure of oxygen (*p*O_2_) (Jones, Rozance, et al., 2019). In addition, HOX fetuses have 25% lower whole body glucose utilization rates compared with normal fetuses (Jones, Rozance, et al., 2019). This suggests a switch in oxidative fuel preference away from glucose and potentially toward alternate substrates in the HOX fetus. Accordingly, we hypothesized that hypoxemia in uteroplacental tissues would increase glycolysis and decrease mitochondrial oxidation of substrates (pyruvate and amino acids). This would supply lactate to the fetus but may alter the allocation of glucose and amino acids to the fetus. To test this, we measured net uteroplacental flux rates for oxygen, glucose, lactate, pyruvate, and amino acids. We also measured molecular targets regulating glucose utilization, pyruvate oxidation, lactate production, and nutrient signaling pathways (AMPK, mTOR) to determine if hypoxemia causes metabolic reprogramming in the placenta.

## 2 METHODS

### 2.1 Ethical approval

All animal procedures were approved by the Institutional Animal Care and Use Committee at the University of Colorado School of Medicine (IACUC Protocol #00465). Pregnant animals were supplied from Nebeker Ranch (Lancaster, CA) and studied at the Perinatal Research Center (Aurora, Colorado). The Perinatal Research Center is accredited by the Association for Assessment and Accreditation of Laboratory Animal Care (AAALAC) and is compliant with United States guidelines, including the Animal Welfare Act and Public Health Service Policy. All experimental work was performed and reported according to the Animal Research: Reporting of In Vivo Experiments (ARRIVE) guidelines (Percie du Sert et al., 2020).

### 2.2 Hypoxemia model in pregnant sheep

The hypoxemia model and animals used in this study have been previously reported (Jones, Rozance, et al., 2019). A brief description and related methodological details are provided. Pregnant Columbia‐Rambouillet ewes carrying singletons were studied. Ewes were fed ad libitum alfalfa pellets (Standlee Hay) and had free access to water. Feed and water intake logs and medical records were maintained daily. Ewes were housed in individual carts during the duration of experimental procedures.

Surgery was performed at ~119 days of gestation (~147 days gestation length) to surgically place indwelling catheters in the maternal and fetal vasculature (Hay et al., 1981, 1984). Ewes were fasted for 24 h prior to surgery. A maternal jugular catheter was placed for administration of diazepam (0.2 mg kg^−1^) and ketamine (17.5 mg kg^−1^) and ewes were then maintained on isoflurane inhalation anesthesia (2%–5%) for the remainder of the surgical procedure. At surgery, procaine penicillin G (600,000 U, i.m.) and ampicillin (500 mg, intra‐amniotically) were administered prior to closing uterine and abdominal incisions. Flunixin meglumine analgesic (Banamine, 2.2 mg kg^−1^, i.m.) and probiotics (Probios, 10 g, oral) were administered for 72 h postoperatively to the ewe. During surgery, the uterus was exteriorized with a mid‐line incision to surgically place indwelling polyvinyl catheters (20G) in the maternal and fetal vasculature (Hay et al., 1981, 1984). Maternal catheters were placed in the femoral artery and femoral vein via a groin incision. Fetal catheters were placed in the common umbilical vein, fetal artery (advanced into the abdominal aorta), and femoral vein (advanced into the inferior vena cava). A uterine vein catheter was placed and advanced into the common vein draining the pregnant uterus. Catheters were filled with 5% heparinized saline and subcutaneously tunneled to the ewe's flank, exteriorized through the skin, and kept in a plastic pouch sutured to the skin. A tracheotomy was performed to place a non‐occlusive catheter (Formulation ND‐100‐65; 13G) in the maternal trachea (Gleed et al., 1986; Harvey et al., 1993; Yates et al., 2012). A vertical skin incision was made below the endotracheal tube cuff and the trachea was cauterized between two cartilaginous rings. The catheter was advanced through the tracheotomy and anchored to the surrounding tissue using suture and the skin incision was sutured closed. The tracheal catheter was subcutaneously tunneled to the ewe’s shoulder and kept in a plastic pouch sutured to the skin. Ewes were allowed to recover for at least 5 days before experimental procedures began.

Beginning at ~125 days of gestation (sheep gestation is ~147 days), ewes were randomly assigned to hypoxemia (HOX; *n* = 11) or control (CON; *n* = 7) groups, after which the study was performed unblinded to maintain fetal experimental conditions. Maternal hypoxemia was induced using tracheal insufflation of humidified nitrogen gas (100% N_2_) to reduce maternal and subsequently fetal arterial *p*O_2_ for ~9 days. Control ewes received humidified compressed air gas (21% O_2_, 78% N_2,_ 1% other trace gases) by tracheal insufflation for the same duration. Daily whole blood samples were collected from the maternal and fetal artery to monitor fetal *p*O_2_, and the rate of N_2_ gas was subsequently adjusted to target and maintain a fetal arterial *p*O_2_ between 12 and 16 mm Hg in the HOX group for the study duration. This fetal *p*O_2_ range was chosen to mimic fetal hypoxemia in age‐matched fetuses with placental insufficiency IUGR (Brown et al., 2015; Thorn et al., 2013). The fetal *p*O_2_ at the end of study in CON and HOX fetuses are shown in Table 3 (Jones, Rozance, et al., 2019). Previously, we reported daily air and nitrogen insufflation rates and maternal and fetal arterial *p*O_2_ measurements across the duration of treatment in CON and HOX groups along with maternal artery, umbilical vein, and fetal artery data on the final day of study for O_2_ content, *s*O_2_, *p*CO_2,_ hematocrit, bicarbonate, pH, glucose and lactate levels, and fetal weight (Jones, Rozance, et al., 2019).

### 2.3 Metabolic nutrient uptake study and tissue collection

Metabolic studies were performed on the final day of treatment (~134 days) to measure uterine and umbilical blood flow and oxygen and nutrient uptake rates (Hay & Meznarich, 1986; Meschia et al., 1966). A 3‐ml bolus of ^3^H_2_O was infused to measure blood flow as previously described (Brown et al., 2017; Meschia et al., 1966; Molina et al., 1991). After 90–120 min, blood was simultaneously sampled from the maternal artery, uterine vein, umbilical vein, and fetal artery four times at 20–30 min intervals to characterize the steady state period. Fetal blood was replaced isovolumetrically with heparinized maternal arterial blood (15 ml h^−1^) throughout the steady state period.

Immediately following the metabolic study, ewes were anesthetized with intravenous (i.v.) diazepam (0.2 mg kg^−1^) and ketamine (17.5 mg kg^−1^) to deliver the fetus via maternal laparotomy and hysterotomy. Subsequently, a lethal dose of sodium pentobarbital (390 mg ml^−1^, Fatal Plus, Vortech Pharmaceuticals) was administered i.v. to euthanize the ewe and fetus. For each animal, fetal weight was recorded and organs were dissected, weighed, and snap frozen in liquid nitrogen. Total uterine weight was measured followed by dissection and measurement of the uterine membrane, uterine tissue, and placentome weight. Individual placentomes were classified into categories (A, B, C, D) based on gross morphological appearance to determine whether hypoxia affected the proportion of placentomes across categories (Vatnick et al., 1991). Representative placentomes (*n* = 3 from each animal) of similar size and type were separated into caruncle or cotyledon sides and snap frozen. Only type A and B were selected as these represent the greatest proportion of placentomes in both CON and HOX groups and allow for similar comparisons between groups that are independent of gross morphology differences. To obtain homogeneous tissue samples for downstream analysis, the cotyledon tissues from each animal were combined and ground in liquid nitrogen.

### 2.4 Biochemical analyses

In all venous and arterial samples, whole blood *p*O_2_ and O_2_ content were measured with the ABL 800 Flex blood gas analyzer (Jones, Rozance, et al., 2019). Plasma glucose and lactate concentrations were measured using the Yellow Springs Instrument model 2900 Select Biochemistry Analyzer. Pyruvate concentrations were determined in deproteinized whole blood samples (Houin et al., 2015; Teng et al., 2002). Plasma amino acids were measured using a Dionex ^TM^ ICS 5000+ high pressure ion chromatograph with Pickering PCX Pinnacle 120—four channel variable wavelength detector for post column derivatization and ultraviolet detection (Thermo Electron North America LLC) (Rozance et al., 2009). Plasma ^3^H_2_O concentrations were measured by liquid scintillation (Houin et al., 2015; Thorn et al., 2013).

### 2.5 Calculations

Uterine and umbilical blood flow were determined by steady‐state diffusion of ^3^H_2_O (Meschia et al., 1966). Uterine uptake rates were calculated using the Fick principle multiplying uterine blood flow by maternal artery‐uterine vein difference in substrate concentration. Umbilical (fetal) uptake rates were calculated using the Fick principle multiplying umbilical blood flow by umbilical vein‐fetal artery difference in substrate concentration (Hay et al., 1981; Meschia et al., 1966; Molina et al., 1991). Net uteroplacental uptake rates were calculated as the difference between uterine minus umbilical uptake absolute rates for oxygen, glucose, and amino acids. Total uteroplacental lactate production was the sum of uterine and umbilical lactate uptake rates. Total uteroplacental pyruvate uptake was the sum of uterine uptake and fetal output. All rates are expressed on an absolute basis without weight adjustments since there were no differences in maternal, uteroplacental, or fetal weights (see Table 2). Analyses also were performed with weight‐specific rates, relative to fetal or placental weight, and there were no differences between those rates and the absolute rates (*data not shown*). Weight‐specific rates of umbilical blood flow, umbilical oxygen uptake, and umbilical glucose uptake were previously reported (Jones, Rozance, et al., 2019), and are reported here again as absolute rates as they are necessary for comparison with uterine uptake rates and for the uteroplacental calculations. All other rates reported herein have not been previously published.

Nutrient‐oxygen metabolic quotients were calculated for glucose, lactate, pyruvate, and amino acids (Battaglia & Meschia, 1978; Hay et al., 1983; Regnault et al., 2013). Umbilical substrate:oxygen quotients were calculated by dividing the whole blood umbilical vein‐fetal artery difference in substrate concentration by the umbilical vein‐fetal artery difference in whole blood O_2_ content, multiplied by the number of oxygen molecules required to oxidize one molecule of substrate. Uterine substrate:oxygen quotients were calculated similarly except using maternal artery‐uterine vein differences. Uteroplacental quotients were estimated using the net uptake of each substrate multiplied by the number of oxygen molecules required and divided by the uteroplacental oxygen utilization rate (Carver & Hay, 1995). Previously, we reported only umbilical metabolic quotients for glucose and lactate (Jones, Rozance, et al., 2019), and we present those data here to use to calculate the sum of all quotients.

Herein, data are reported for 7 CON and 11 HOX maternofetal units (Jones, Rozance, et al., 2019), unless otherwise noted in figure legends. Due to umbilical venous catheter failures, umbilical data were included for 7 CON and 8 HOX animals. Due to uterine venous catheter failures, uterine data were included for 6 CON and 7 HOX animals. For net uteroplacental data, there were 6 CON and 4 HOX animals with all four sampling catheters patent. Amino acid measurements were not available for one HOX animal for the uterine and uteroplacental data. To complement the measured uteroplacental flux rates in the CON and HOX groups, uteroplacental flux rates also were estimated using the mean values for uterine and umbilical data obtained from the full cohort (*as presented in* Figure 6). The respective relative contributions of uterine, uteroplacental, and fetal nutrient allocation were calculated.

### 2.6 Gene expression

RNA was isolated from placental cotyledon tissue, reverse transcribed to cDNA, and qPCR was performed as previously described and following MIQE guidelines (Bustin et al., 2009; Jones, Brown, et al., 2019; Rozance et al., 2020). Primers were used as previously reported or new primers were designed to span exon regions within a gene (Table 1). The geometric mean of five reference genes was calculated with *ACTB*, *PPIB*, *GAPDH*, *RPS15*, and *HPRT1* (as *defined in* Table 1) and used to normalize qPCR results. Data are expressed relative to the CON group by calculating by dividing the expression for each sample relative to the mean in the CON group.

**TABLE 1:** Reagent list

Common gene name	Symbol	Forward primer	Reverse primer
Cytochrome c oxidase subunit 4I1	*COX4I1*	TTTCCACCTCGGTGTGTGTT	TAGTCACGCCGGTCCACATA
Cytochrome c oxidase subunit 4I2	*COX4I2*	GTCCTTCAGAGCTGCCTGG	CGGTACTTCCTGGGGTGTG
Solute carrier family 2 member 1	*GLUT1*	TGGGAGGCATGATTGGTTCC	TGAGAAGCCCATGAGCACAG
Solute carrier family 2 member 4	*GLUT4*	AGCAGCTGTCAGGCATCAAT	CCGATGGTAGCATAGGCTGG
Pyruvate dehydrogenase compelx component X	*PDH*	GTTAAGGGGGCTGCTAGGTG	AGCCACTGCGTACTGTGAAA
Pyruvate dehydrogenaes kinase 1	*PDK1*	TGGAGCATCACGCTGACAAA	CTCAGAGGAACACCACCTCC
Pyruvate dehydrogenase kinase 2	*PDK2*	TACATGGCCTCTCCTGACCT	AAGCATGTGGTAGAGGTGGG
Pyruvate dehydrogenase kinase 4	*PDK4*	CCCAGAGGACCAAAAGGCAT	GGGTCAGCTGTACAGGCATC
Phosphofructokinase, liver isoform	*PFK1*	TGGTGGCTCCATGCTGGGGA	GCAGGGCGTGGATGCTGTGA
Solute carrier family 16 member 1	*MCT1*	GTGGCTTGATTGCTGCTTCC	GCCAATCATGGTCAAAGCCG
Lactate dehydrogenase A	*LDHA*	CATGGCCTGTGCCATCAGTA	GGAAAAGGCTGCCATGTTGG
Lactate dehydrogenase B	*LDHB*	GAGGGAGCGATCCCAAACAA	CAGAATGCTGATGGCACACG
Succninate dehydrodgenase, b	*SDHB*	AGAGACGACTTCACGGAGGA	AGCTTTCCCAGGATTCAGCC
Pyruvate kinase, muscle, isoform 1	*PKM1*	GTGTTTAGCGGCAGCTTTGA	CTGTCTGGTGATTCCGGGTC
Pyruvate kinase, muscle, isoform 2	*PKM2*	GGGCCATAATCGTCCTCACC	CTGTCTGGTGATTCCGGGTC
Pyruvate kinase, liver	*PKLR*	TGGCGGGAAAGCCCGTTGTC	CCAGAACGGCGTTGGCCACA
Ribosomal proten S15	*S15*	ATCATTCTGCCCGAGATGGTG	CGGGCCGGCCATGCTTTACG
Cyclophilin B	*PPIB*	GCCTTGGCTACAGGAGAGAA	GGGAAGCGTTCACCGTAGAT
Mitochondrial pyruvate carrier, 2	*MPC2*	TAAAGTGGAGCTCCTGCTGC	ATGTCAGCCAATCCAGCACA
Mitochondrial pyruvate carrier, 1	*MPC1*	TCGGAACTGGCTCCTGTTTG	GCCGGTTCTTCATCTCCCAT
Hypoxanthine phosphoribosyltransferase 1	*HPRT1*	AGCGTGGTGATTAGCGATGA	CACATCTCGAGCCAGTCGTT
Actin, beta	*ACTB*	TGCAGAAAGAGATCACTGCC	GACAGCGAGGCAGGATGG
Glyceraldehyde phosphate dehydrogenase	*GAPDH*	TGGAGGGACTTATGACCACTG	TAGAAGCAGGGATGATGTTCT
*Antibody*	*Dilution*	*Supplier (Catalog #); RRID*	*Validated reference*
P‐PDH (S293)	1:1000	Abcam (92696); AB_10711672	Pendleton et al. (2019)
PDH	1:2000	Abcam (110330); AB_10858459	Pendleton et al. (2019)
LDHA	1:1000	Abcam (47010); AB_1952042	
ph‐AMPK (Thr172)	1:1000	Cell Signaling (2531S); AB_330330	Rozance et al. (2020)
AMPK alpha	1:1000	Cell Signaling (2793S); AB_915794	Rozance et al. (2020)
P‐mTOR (Y2448)	1:1000	Cell Signaling (2971); AB_330970	Jones, Brown, et al. (2019)
mTOR	1:1000	Cell Signaling (4517); AB_1904056	Jones, Brown, et al. (2019)
P‐S6 (S235/6)	1:1000	Cell Signaling (2211); AB_331679	Jones, Brown, et al. (2019)
S6	1:1000	Cell Signaling (2317); AB_2238583	Jones, Brown, et al. (2019)
P‐4E‐BP1 (Thr37/46)	1:1000	Cell Signaling (9455); AB_330949	Brown et al. (2009)
4E‐BP1	1:1000	Cell Signaling (9452); AB_331692	Brown et al. (2009)

### 2.7 Protein expression

Whole cell lysates were prepared from placental cotyledon tissue using buffer and western immunoblotting was performed as previously described (Jones, Brown, et al., 2019; Rozance et al., 2020). The antibodies used are provided in Table 1 and include references to previous studies that have verified their specificity in ovine samples, when available (Brown et al., 2009; Jones, Brown, et al., 2019; Pendleton et al., 2019; Rozance et al., 2020). Briefly, 30 µg protein was loaded with 4X DTT (1 M) in equal volumes of buffer, separated on a 4%–12% polyacrylamide gels and transferred onto nitrocellulose membranes (Bio‐Rad). Antibody specificity was verified by the presence of a single band at the expected molecular weight. Bands for phosphorylated and total forms of a protein were verified to be of similar size based on migration in gels when blot images were aligned. Protein bands were visualized using IR‐Dye IgG secondary antibody (LI‐COR) and protein expression quantified with Image Studio (LI‐COR). Samples were run on two blots (nine samples each plus a reference sample on each gel to account for gel‐to‐gel differences). Target band densities were normalized to the reference samples on each blot. For phosphorylated proteins, data are expressed as a ratio of phosphorylation to total expression, in addition to absolute levels of phosphorylated and total protein expression. Before blocking and antibody incubations, the equality of sample loading was measured using the Total Protein Stain (LI‐COR). LDHA protein expression is expressed relative to the total protein stain quantification. Data are presented as a fold change relative to the mean of the CON group.

### 2.8 Pyruvate dehydrogenase enzyme activity

Activity of PDH was measured in placental tissue (40 mg) homogenized in 400 μl ice‐cold PDH Assay Buffer (MAK183, Sigma Aldrich). Protein concentrations were determined using a Pierce BCA Protein Assay (ThermoFisher Scientific) and 10 μg protein was loaded per reaction in duplicate. Assays were performed at 37℃ and A_450_ was measured every 5 min for 30 min. The Δ A_450_ was calculated for 15 min of the linear reaction and is proportional to the NADH concentration produced by PDH enzymatic reaction converting pyruvate into acetyl CoA.

### 2.9 Lactate dehydrogenase enzyme activity

Placental tissue (50 mg) was homogenized in 500 μl ice‐cold CelLytic MT Buffer (Sigma‐Aldrich). LDH activity was assessed using the LDH Activity Assay (ab102526, Abcam). Protein concentrations were determined as described above and 1 μg protein was loaded per reaction in duplicate. Assays were performed at 37℃ and A_450_ was measured every 5 min for 40 min. The Δ A_450_ was calculated for 20 min of the linear reaction between 20‐ and 40‐min time points and is proportional to the NADH concentration produced by the LDH enzymatic reaction converting lactate into pyruvate. Results were normalized to the amount of protein loaded in the reaction.

### 2.10 Thiobarbituric acid‐reactive substances

TBARS content was measured colorimetrically (no. 700870; Cayman) in placental tissue protein lysate samples prepared as described for western blotting (Rozance et al., 2020). Results are expressed relative to protein content.

### 2.11 Statistical analysis

Data were analyzed by unpaired student's *t*‐test or Mann–Whitney *U*‐test, when variances were different between groups (as indicated in Table 3 for fetal arterial lactate and pyruvate concentrations), and linear regression and correlation analyses were performed using GraphPad Prism 9.0 (GraphPad Software). The analysis used is indicated in the figures and tables. Data are presented as mean ± SD. Statistical differences are declared at *p* ≤ 0.05. Fetal sex effects were evaluated using a two‐way ANOVA with fixed effects of treatment (CON, HOX) and fetal sex. No significant effects of fetal sex were found and because of the small sample size for some variables, fetal sex was not included in the final analyses and data from female and male fetuses were combined. Individual values are denoted in all figures containing in vivo data using triangles for male and circles for female fetuses. For data reported in tables, data are available in a public repository (https://figshare.com/s/09777109e6fe5b4cf13d).

## 3 RESULTS

### 3.1 Maternal and fetal characteristics during sustained hypoxemia

**FIGURE 1:**
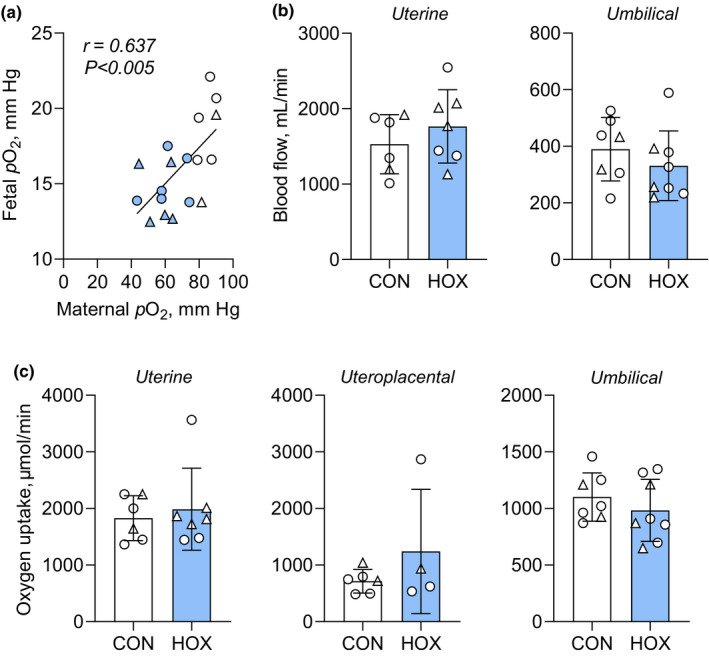
Effects of sustained hypoxemia on blood flow and oxygen utilization. (a) Relationship between maternal and fetal arterial *p*O_2_ measured after 9 days of sustained hypoxemia in CON (white symbols) and HOX (blue symbols) groups. Linear regression was performed, and the Pearson correlation coefficient (*r*) and significance are indicated. (b) Uterine blood flow (*p *= 0.361) and umbilical blood flow (*p *= 0.358) were measured in CON and HOX groups. (c) Uterine (*p *= 0.643), uteroplacental (*p *= 0.272), and umbilical (*p *= 0.376) oxygen utilization (net uptake) rates. Female fetuses are shown with circle and male fetuses with triangle symbols. Means ± SD shown. All results were analyzed by *t*‐test. Weight specific rates of umbilical blood flow and umbilical oxygen uptake were previously reported (Jones, Rozance, et al., 2019)

Maternal and fetal blood oxygen concentrations and *p*O_2_ were decreased and maintained for 9 days in HOX compared to CON groups (Jones, Rozance, et al., 2019) (Table 3). Maternal and fetal *p*O_2_ were positively correlated (Figure 1a). Maternal body weight, total uterine weight (including all placentomes and membranes), and placental weight (sum of all placentomes) was similar between HOX and CON (Table 2). Placentas from HOX pregnancies had fewer total placentomes, explained by fewer class B placentomes (Table 2). Fetal weight and fetal: placental weight was not different between CON and HOX groups (Jones, Rozance, et al., 2019) (Table 2).

**TABLE 2:** Uteroplacental morphometrics and fetal weight

Variable	CON	HOX	*p* value
Maternal body weight, kg	58.6 ± 6.7	61.0 ± 8.0	0.540
Uteroplacenta, total, g	1982.0 ± 562.3	1883.1 ± 587.8	0.743
Uterus, g	580.0 ± 75.3	658.7 ± 101.8	0.116
Uteroplacenta membrane, g	432.2 ± 101.8	416.1 ± 72.3	0.716
Placental weight, g	304.0 ± 68.5	262.7 ± 58.9	0.218
Placentome number, total	84.7 ± 7.0	62.9 ± 24.1	0.043
Placentome A type, number	4.4 ± 3.3	8.6 ± 9.1	0.294
Placentome B type, number	64.4 ± 22.8	39.0 ± 20.7	0.040
Placentome C type, number	7.7 ± 3.6	11.7 ± 7.0	0.215
Placentome D type, number	7.3 ± 13.6	4.9 ± 7.9	0.675
Average mass per placentome, g	3.7 ± 1.1	4.9 ± 2.1	0.190
Fetal body weight (*), g	3049.1 ± 349.9	2953.3 ± 573.9	0.713
Fetal: Placental weight ratio, g/g	10.3 ± 1.3	11.4 ± 1.6	0.126
Male:Female ratio	2:5	6:5	

*n* = 7 CON, 11 HOX.

Values are mean ± SD.

* Previously reported in Jones, Rozance, et al. (2019).

### 3.2 Blood flow and uteroplacental oxygen utilization

Uterine blood flow rates and umbilical blood flow rates (Figure 1b) were similar between CON and HOX groups. The rate of oxygen uptake across the uterine circulation, supplying oxygen to the uteroplacental and fetal tissues, was similar between CON and HOX groups (Figure 1c). Uteroplacental oxygen utilization, calculated as the difference between the absolute rates of uterine and umbilical uptake, also was similar between CON and HOX groups (Figure 1c). There was no difference in fetal oxygen utilization rates (Figure 1c).

### 3.3 Glucose, lactate, and pyruvate concentrations and net uteroplacental flux

**FIGURE 2:**
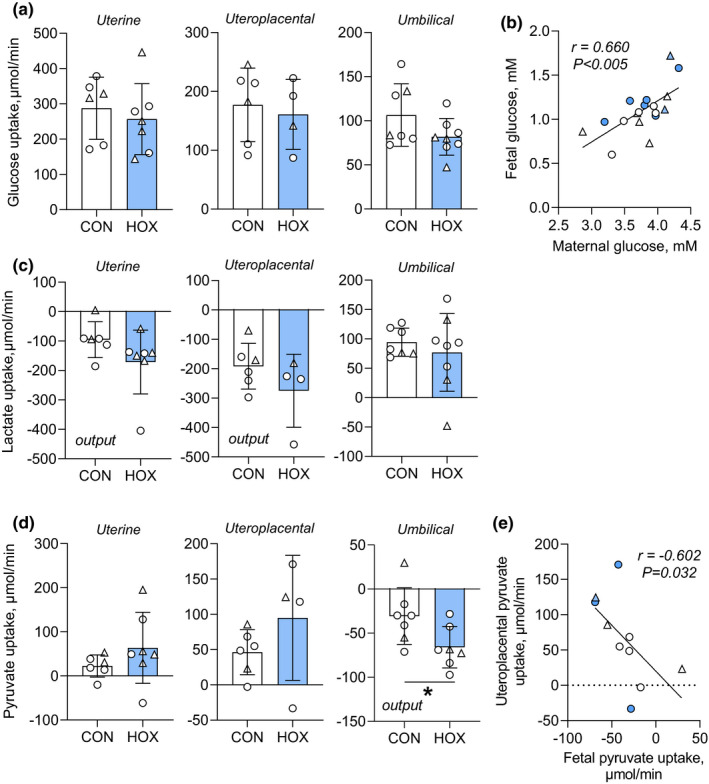
Effect of sustained hypoxemia on net uterine, uteroplacental, and umbilical glucose, lactate, and pyruvate flux. (a) Uterine (*p *= 0.573), uteroplacental (*p =* 0.694), and umbilical (*p *= 0.118) net glucose uptake rates in CON and HOX groups. (b) Relationship between fetal and maternal arterial whole blood glucose concentrations measured with linear regression in CON (white symbols) and HOX (blue symbols) groups. (c) Uterine (*p *= 0.160), uteroplacental (*p *= 0.223), and umbilical (*p *= 0.523) net lactate uptake rates in CON and HOX groups. (d) Uterine (*p *= 0.256), uteroplacental (*p *= 0.244), and umbilical (*, *p *= 0.037) net pyruvate uptake rates in CON and HOX groups. Negative uptake rates indicate net output. (e) Relationship uteroplacental net pyruvate uptake and umbilical net pyruvate uptake rates measured with linear regression in CON (white symbols) and HOX (blue symbols) groups. Female fetuses are shown with circle and male fetuses with triangle symbols. Means ± SD shown. All results were analyzed by *t*‐test. Pearson correlation coefficients (*r*) and significance are shown for regression analyses. Weight specific rates of umbilical glucose uptake were previously reported (Jones, Rozance, et al., 2019)

The net uterine uptake rate of glucose, supplying both uteroplacental and fetal tissues, was similar between CON and HOX groups (Figure 2a). Net uteroplacental and fetal uptake rates of glucose also were similar between CON and HOX groups (Figure 2a). In addition, maternal and fetal glucose concentrations were similar between CON and HOX groups (Table 3). There also was a positive relationship between fetal and maternal glucose concentration across both groups (Figure 2b), supporting similar placental glucose transport.

**TABLE 3:** Maternal and fetal arterial nutrient concentrations

	Maternal artery			Fetal artery		
Nutrient	CON	HOX	*p* value	CON	HOX	*p* value
*p*O_2_, mmHg (*)	84.91 ± 4.74	59.2 ± 10.0	<0.001	18.39 ± 2.86	14.66 ± 1.781	0.004
Oxygen, mM (*)	4.9 ± 0.52	4.3 ± 0.66	0.071	2.43 ± 0.39	1.55 ± 0.57	0.003
Carbohydrates (mM)						
Glucose (*)	3.78 ± 0.27	3.75 ± 0.41	0.897	0.98 ± 0.24	1.17 ± 0.26	0.133
Lactate (*)	0.83 ± 0.24	0.93 ± 0.21	0.406	2.11 ± 0.38	7.04 ± 6.20	0.002**
Pyruvate	0.11 ± 0.03	0.09 ± 0.03	0.238	0.12 ± 0.04	0.23 ± 0.10	0.002**
Essential amino acids (nM)						
HIS	41.4 ± 7.0	43.7 ± 8.0	0.535	45.1 ± 13.6	49.8 ± 16.6	0.544
ILEU	99.4 ± 15.3	105.5 ± 22.0	0.534	76.8 ± 19.5	92.2 ± 24.4	0.179
LEU	122.9 ± 20.7	124.4 ± 29.7	0.914	120.2 ± 28.4	133.9 ± 42.0	0.461
LYS	105.5 ± 11.9	121.1 ± 22.1	0.107	64.5 ± 17.5	75.0 ± 29.1	0.407
MET	27.2 ± 7.0	28.5 ± 5.4	0.669	93.9 ± 27.2	89.0 ± 21.5	0.679
PHE	49.5 ± 7.2	50.4 ± 9.1	0.832	100.7 ± 17.2	109.7 ± 34.9	0.539
THR	88.1 ± 27.4	111.8 ± 54.8	0.306	168.0 ± 70.0	200.0 ± 76.1	0.384
TRP	28.1 ± 5.8	29.6 ± 8.8	0.715	33.0 ± 6.5	37.3 ± 6.5	0.186
VAL	195.5 ± 34.9	209.6 ± 59.3	0.581	351.3 ± 99.0	372.1 ± 104.8	0.680
Non‐essential amino acids (nM)						
ALA	112.5 ± 16.6	135.3 ± 27.7	0.068	291.9 ± 24.9	410.3 ± 128.1	0.029
ARG	132.5 ± 40.9	141.7 ± 39.0	0.638	76.2 ± 18.6	58.9 ± 30.1	0.193
ASPG	30.8 ± 8.8	35.6 ± 12.7	0.398	36.2 ± 7.4	46.2 ± 14.4	0.112
ASP	7.4 ± 2.0	8.5 ± 2.4	0.317	31.3 ± 6.5	21.3 ± 6.1	0.004
CYS	22.4 ± 3.4	27.4 ± 6.6	0.082	17.4 ± 4.0	15.1 ± 2.5	0.148
GLU	50.1 ± 8.0	53.0 ± 10.5	0.545	45.7 ± 15.8	27.0 ± 9.8	0.007
GLN	205.6 ± 20.2	240.8 ± 40.4	0.049	373.1 ± 40.2	423.4 ± 83.3	0.158
GLY	299.1 ± 72.6	311.1 ± 76.2	0.745	365.7 ± 99.0	286.0 ± 98.7	0.115
ORNI	56.9 ± 16.0	72.5 ± 30.2	0.231	48.1 ± 12.7	41.3 ± 10.3	0.228
PRO	60.9 ± 12.3	82.9 ± 32.3	0.107	121.9 ± 22.4	189.2 ± 110.6	0.135
SER	50.4 ± 11.5	57.5 ± 24.3	0.480	657.1 ± 105.4	398.2 ± 183.0	0.004
TAU	47.8 ± 26.5	59.9 ± 19.1	0.275	57.1 ± 31.7	82.6 ± 41.2	0.183
TYR	55.6 ± 12.3	65.1 ± 19.8	0.277	86.3 ± 15.9	130.6 ± 38.5	0.011

*n* = 7 CON, 11 HOX.

Values are mean ± SD.

* Previously reported in Jones, Rozance, et al. (2019).

** Mann whitney test for non‐parametric data.

Uteroplacental tissues produce lactate that is released into the maternal and fetal compartments. The net uteroplacental flux of lactate into the uterine circulation and to the mother was not different between groups (Figure 2c). Uteroplacental lactate output (total production), calculated as the sum of uterine output to the mother plus net uptake by the fetus, was not different in HOX compared to CON groups (Figure 2c). There were no differences in the net fetal uptake rate of lactate produced by the uteroplacental tissues between CON and HOX fetuses (Figure 2c). In HOX fetuses, however, lactate concentrations were increased over threefold (Table 3), yet there was no difference in maternal lactate concentrations.

Pyruvate is taken up and utilized by uteroplacental tissues from the maternal (uterine) and fetal (umbilical) circulations. The net uptake rate of pyruvate across the uterine circulation to uteroplacental tissues was similar between CON and HOX groups (Figure 2d). The rate of uteroplacental pyruvate uptake, calculated as the sum of uterine uptake and fetal output, was not increased in HOX compared to CON (Figure 2d). The net fetal output rate of pyruvate to uteroplacental tissues was increased over twofold in the HOX fetus (Figure 2d) and positively correlated with uteroplacental pyruvate uptake (Figure 2e). Further, pyruvate concentrations were twofold higher in the HOX compared to CON fetuses, with no difference in maternal concentrations (Table 3).

### 3.4 Amino acid concentrations and net uptake rates

**FIGURE 3:**
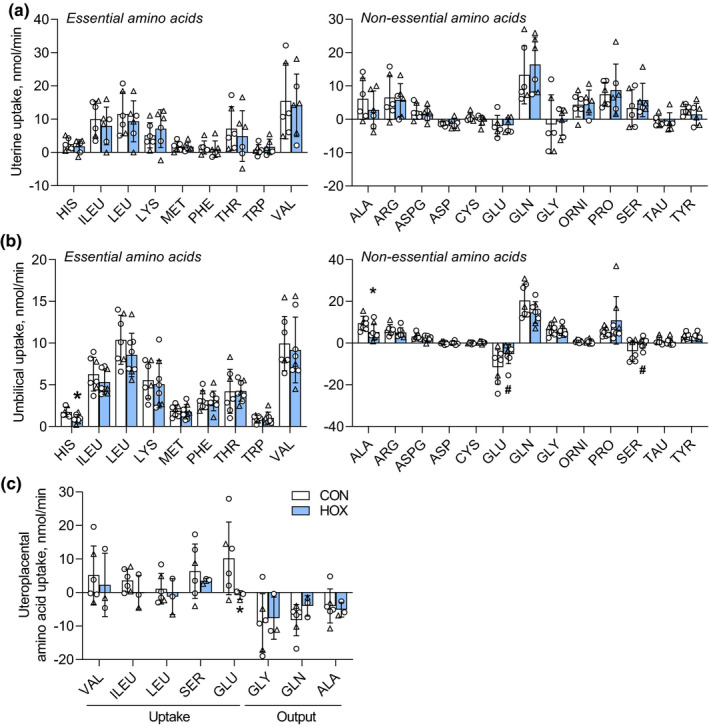
Effect of sustained hypoxemia on net amino acid flux rates. (a) Uterine net amino acid uptake rates in CON (*n* = 6) and HOX (*n* = 6) groups (all *p *> 0.05). (b) Umbilical net amino acid uptake rates in CON (*n* = 6) and HOX (*n* = 6) (#, *p *< 0.15; *, p < 0.05). (c) Net uteroplacental uptake rates of selected amino acids in CON (*n* = 6) and HOX (*n* = 3) (*, *p *< 0.05). Means ± SD shown. All results were analyzed by *t*‐test

Maternal amino acid concentrations were similar between CON and HOX groups, except for increased Glu in the HOX group (Table 3). HOX fetuses had lower plasma concentrations of aspartate, serine, and glutamate and higher concentrations of alanine and tyrosine compared to CON (Table 3). The net uptake rate across the uterine circulation for each of the individual amino acids, supplying uteroplacental and fetal tissues, was not different between groups (Figure 3a). HOX fetuses had decreased net uptake of alanine and histidine and trends (*p *< 0.15) for decreased net output of serine and glutamate (Figure 3b), two amino acids that are normally released by the fetus (Battaglia et al., 2003). Given the small concentrations differences and extraction coefficients across the uteroplacental circulation, in addition to the complexity of amino acid metabolism with transamination, degradation, and/or synthesis in the placenta (Brown, Regnault, et al., 2017; Cetin, 2001; Hay, 1991b; Vaughan & Fowden, 2016), we focused on specific amino acids that are expected to have significant net uptake or release by uteroplacental tissues (Cetin, 2001). In the CON group, there was a net uptake of glutamate by uteroplacental tissues (Figure 3c). In contrast, in the HOX group, there was net uteroplacental output of glutamate (Figure 3c). There were no differences on net uteroplacental uptake of valine, isoleucine, leucine, and serine nor the net output of glycine, glutamine, and alanine between CON and HOX groups (Figure 3c).

### 3.5 Nutrient allocation to the fetus and utero‐placenta

Nutrient‐oxygen metabolic quotients provide a flow‐independent measure of net nutrient uptake and represent a proxy for the fraction of oxygen uptake required for complete substrate (carbon) oxidation. A sum of metabolic quotients (glucose, amino acids, pyruvate, plus lactate) equal to 1.0 indicates that net carbon uptake for those substrates is matched with oxygen uptake and is sufficient to support oxidative metabolism. Values greater than 1, support that additional carbon sources are available for growth beyond fueling oxidative metabolism; values less than 1, indicate that exogenous carbon sources alone are not sufficient to fuel oxidative metabolism and growth. There were no differences in the metabolic quotients across the uterine circulation (Table 4). In the HOX fetus, the relative pyruvate:oxygen quotient was larger and negative, demonstrating fetal output (Table 4). There were no differences in the glucose, amino acid (sum), or lactate:oxygen quotients. The sum of all substrate quotient across the umbilical circulation was not different between groups. Uteroplacental metabolic quotients were estimated and suggest a lower glucose and higher pyruvate quotient in HOX uteroplacental tissues, with lesser effects on lactate and amino acid quotients (Table 4).

**TABLE 4:** Substrate:oxygen metabolic quotients

	CON	HOX	*p* value
Uterine^a^			
Glucose:oxygen quotient	0.93 ± 0.18	0.82 ± 0.35	0.31
Lactate:oxygen quotient	−0.16 ± 0.09	−0.25 ± 0.08	0.39
Pyruvate:oxygen quotient	0.04 ± 0.04	0.09 ± 0.13	0.68
Amino acid:oxygen quotient	0.30 ± 0.19	0.39 ± 0.29	0.44
Metabolic quotient sum^c^	1.12 ± 0.12	1.04 ± 0.18	0.74
Umbilical (net fetal)^b^			
Glucose:oxygen quotient (*)	0.57 ± 0.08	0.52 ± 0.08	0.30
Lactate:oxygen quotient (*)	0.26 ± 0.06	0.27 ± 0.09	0.75
Pyruvate:oxygen quotient	−0.09 ± 0.09	−0.19 ± 0.05	<0.05
Amino acid:oxygen quotient	0.44 ± 0.09	0.49 ± 0.11	0.24
Metabolic quotient sum^c^	1.18 ± 0.08	1.06 ± 0.07	0.29
Uteroplacental^d^			
Glucose:oxygen quotient	1.49	1.05	
Lactate:oxygen quotient	−0.78	−0.74	
Pyruvate:oxygen quotient	0.22	0.39	
Amino acid:oxygen quotient	0.18	0.10	
Metabolic quotient sum^c^	1.11	0.79	

^a^ Uterine quotients measureds in *n* = 6 CON, 7 HOX.

^b^ Umbilical quotients measured in *n* = 7 CON, 7 HOX.

^c^ Sum of quotients for glucose, lactate, amino acids, pyruvate.

^d^ Estimated using the mean values from uterine and umbilical data sets.

* Previously reported in Jones, Rozance, et al. (2019).

### 3.6 Effect of hypoxemia on metabolic reprogramming and nutrient sensing in the placenta

**FIGURE 4:**
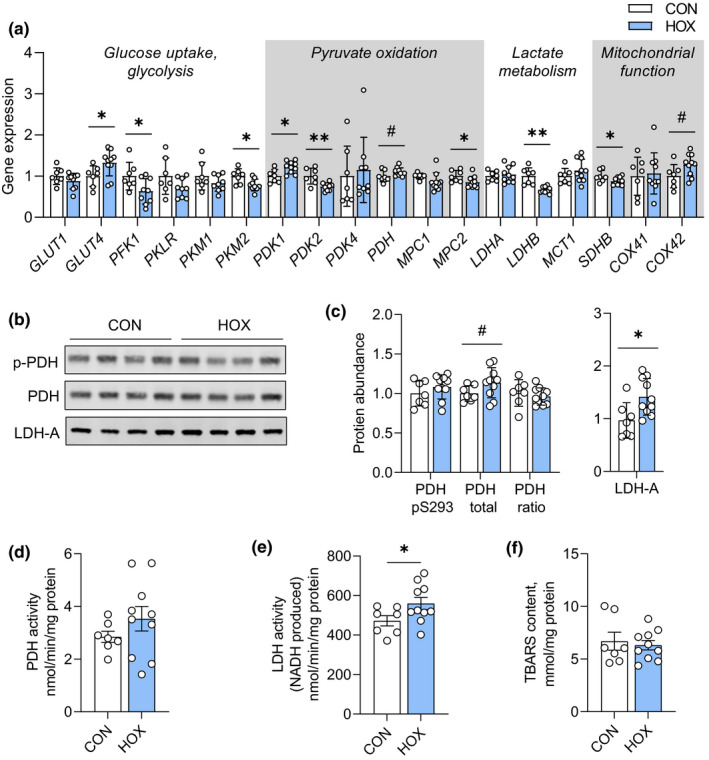
Effect of sustained hypoxemia on pathways regulating metabolic reprogramming in the placenta. (a) Relative expression of genes for glucose uptake and utilization, pyruvate oxidation, lactate metabolism, and mitochondrial function were measured in CON (*n* = 7) and HOX (*n* = 11) placental tissue (cotyledon). #*p *< 0.15, *p < 0.05, **p < 0.01. (b) Protein expression was measured by western blotting in CON (*n* = 7) and HOX (*n* = 11) placental tissue lysates and quantified. A representative blot of each protein is shown. (c) Protein abundance of phosphorylated (*p *= 0.294), total (#,*p *= 0.11), and the ratio of phosphorylated: total PDH (*p *= 0.517). (d) Protein expression of LDH‐A (**, *p *= 0.018). (d) PDH activity measured in placental tissue (*p *= 0.261). (e) LDH activity measured in placental tissue (*, *p *= 0.043). (f) Thiobarbituric acid‐reactive substances (TBARS) measured in placental tissue (*p *= 0.672). Means ± SD are shown. Data were analyzed by *t*‐test

To corroborate the in vivo uteroplacental nutrient flux rates and identify mechanisms regulating uteroplacental carbohydrate metabolism and nutrient signaling during sustained hypoxemia, we compared CON and HOX placental tissue samples. Placentas from HOX pregnancies had increased *GLUT4* gene expression, yet decreased expression of the glycolytic genes, *PFK1* and *PKM2*, and no difference in expression of *PKLR*, *PKM1*, or *GLUT1* (Figure 4a). Expression of *PDK1* was increased, *PDK2* and *LDHB* were decreased, and there was no difference *PDK4* or *LDHA* in HOX compared to CON placentas. Further, *MPC2* and *SDHB* expression was decreased and expression of *COX4I2* was increased. There was no difference in expression of the phosphorylated form (inactive form) or total abundance of PDH (Figure 4b,c) or the activity of the PDH enzyme (Figure 4d). Protein expression of LDHA, however, was increased by 40% in HOX compared to CON placentas (Figure 4b,c). The activity of LDH in HOX placental tissue also was increased (Figure 4e). Placental tissue content of TBARS, a product of oxidative stress, was not different between groups (Figure 4f).

**FIGURE 5:**
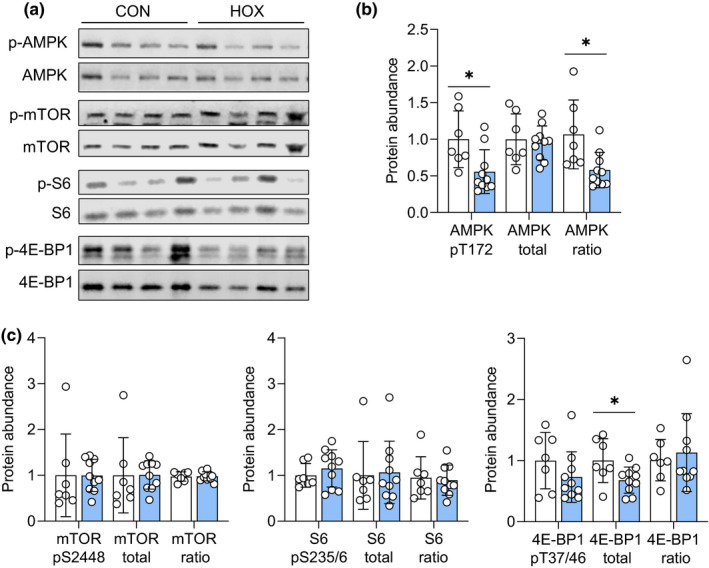
Effect of sustained hypoxemia on nutrient sensing and signaling pathways in the placenta. Protein abundance was measured by western blotting in CON (*n* = 7) and HOX (*n* = 11) placental tissues. (a) Representative western blot image are shown. (b) Protein abundance of phosphorylated (*, *p* = 0.017), total (*p* = 0.730), and the ratio of phosphorylated: total AMPK (*, *p* = 0.013). (c) Protein abundance of phosphorylated (*p *= 0.991), total (*p *= 0.975), and the ratio of phosphorylated: total mTOR (*p *= 0.928). Protein abundance of phosphorylated (*p *= 0.401), total (*p *= 0.855), and the ratio of phosphorylated: total S6 (*p *= 0.786). Protein expression of phosphorylated (*p *= 0.226), total (*p *= 0.648), and the ratio of phosphorylated: total 4E‐BP1 (*, *p *= 0.035). Means ± SD are shown. Data were analyzed by *t*‐test

Expression of phosphorylated AMPK (active) was decreased by 45%, with no difference in total abundance of AMPK protein (Figure 5a,b). There were also no differences in the phosphorylation of mTOR, or its downstream target S6 and 4E‐BP1 (Figure 5a,c). Total protein expression of mTOR and S6 also were similar between groups, yet 4E‐BP1 expression was 30% lower.

## 4 DISCUSSION

Our results provide new mechanisms for how hypoxemia during late gestation reprograms uteroplacental nutrient metabolism and the allocation of nutrients to the fetus. Our in vivo uteroplacental flux data provide evidence for a fetal‐uteroplacental shuttle during sustained hypoxemia whereby uteroplacental tissues produce lactate as fuel for the fetus using pyruvate released from the fetus, rather than pyruvate produced from glucose in the placenta given the absence of increased uteroplacental glucose uptake and glycolytic gene activation. In addition, placental expression and activity levels of metabolic enzymes support that hypoxemia produces changes in uteroplacental nutrient flux and induces metabolic reprogramming. This includes evidence for hypoxic responses that increase mitochondrial efficiency and increase lactate production from pyruvate in the placenta, both features of metabolic reprogramming, but without evidence for an effect on increased glucose utilization and glycolytic flux. The presence of a pyruvate‐lactate shuttle between the placenta and fetus, and other amino acid shuttles (serine‐glycine and glutamate‐glutamine), may provide metabolic advantages for both uteroplacental and fetal tissues. In addition, given the altered proportions of substrates from the uteroplacental, the HOX fetus may develop a switch in substrate preference that enables it to maintain oxidative metabolism (Jones, Rozance, et al., 2019).

### 4.1 Uteroplacental nutrient flux and distribution summary

**FIGURE 6:**
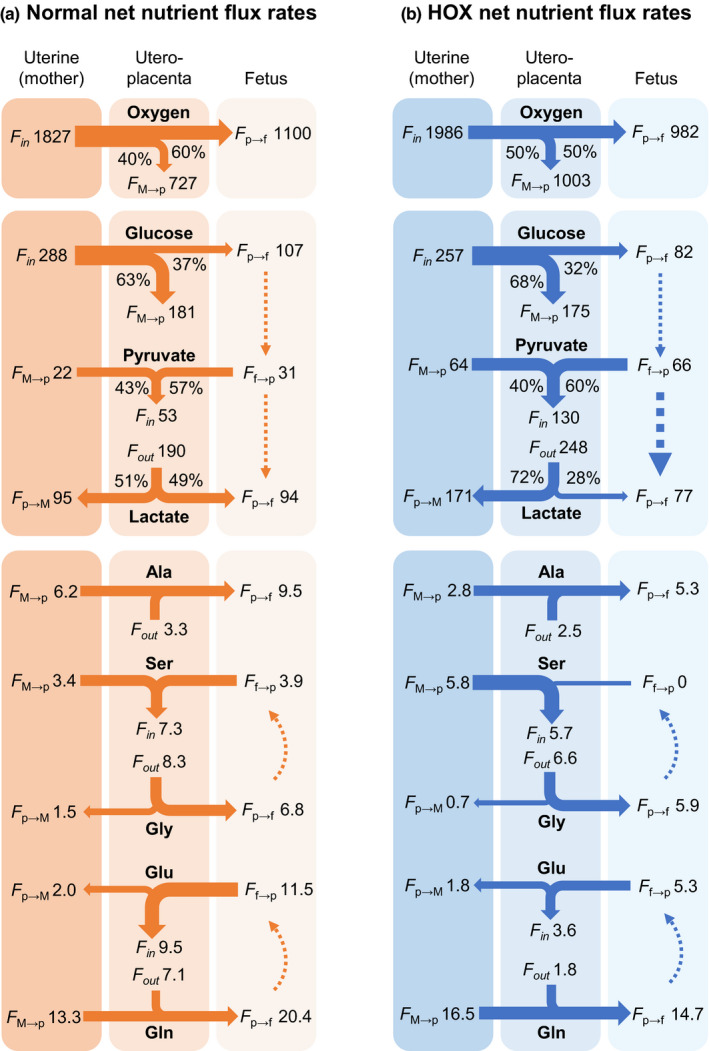
Summary of net uteroplacental nutrient flux rates and relative nutrient allocation. Flux rates for oxygen, glucose, lactate, pyruvate, and selected amino acids are shown in (a) CON and (b) HOX groups. The mean values (absolute rates) for nutrient and oxygen uptake rates across the uterine and umbilical circulation are shown and were used to calculate uteroplacental rates. The solid lines indicate relative magnitude of flux rate and arrows indicate the direction of flux. Dashed arrows indicate a potential fetal conversion of substrates. Relative nutrient allocation as a percentage of total is shown for oxygen, glucose, lactate, and pyruvate with respect to uteroplacental flux (*F*_p_) and distribution to the maternal (*F*_M_) and fetal (*F*_f_) compartments. *F*_in_ represents total flux of a substrate taken up by the placenta. *F*_out_ represents total flux of a substrate out of the placenta

A summary of the net uteroplacental flux rates and relative distribution (percentage) of nutrients, in relation with flux between the maternal and fetal compartments, in CON and HOX groups is shown in Figure 6. The relative flux and distribution of oxygen and glucose from the mother to the utero‐placenta and fetus was similar between CON and HOX groups. Total uteroplacental pyruvate uptake was higher, with similarly higher flux rates from both the maternal and fetal compartments. The relative uteroplacental flux of lactate to the mother was higher in the HOX group. The net uteroplacental flux of alanine from the mother and to the fetus was lower. Net uteroplacental serine flux from the fetus was near zero, with similar glycine flux rates. The uteroplacental flux of glutamate from the fetus was lower in HOX, as was the uteroplacental flux of glutamine to the fetus. The significance of these uteroplacental flux differences is discussed below. We also acknowledge that a limitation of the measured uteroplacental flux rates due to catheter failure is the smaller sample since these rates require functional uterine and umbilical catheters. Thus, in Figure 6, we present uteroplacental flux rates calculated using the mean values for uterine and umbilical rates, which were measured in larger sets of animals and are consistent with the results of directly calculated uteroplacental flux rates in the smaller set of animals.

#### 4.1.1 Carbohydrate metabolism

Previous work in human pregnancies have proposed that during hypoxemia, the placenta increases glucose consumption (glycolysis) via mechanisms resembling metabolic reprogramming (Zamudio et al., 2010). If there was metabolic reprogramming in the placenta of HOX pregnancies, we would expect decreased placental oxygen uptake, increased placental glucose uptake, increased lactate release to the fetus and mother, and decreased glucose supply to the fetus (Illsley et al., 2010; Zamudio et al., 2010). However, given the absence of decreased uteroplacental oxygen consumption or increased glucose utilization (uptake and glycolytic genes), our data only support the increased lactate‐production feature of metabolic reprogramming in the placenta, in addition to mitochondrial effects including decreased *SDHB* and increased *COX42*, which are expected to increase mitochondrial substrate oxidation (Illsley et al., 2010). The gene expression of *GLUT4* was increased in the HOX placenta, however, the function of increased *GLUT4* is not clear since it mediates insulin‐stimulated glucose transport and placental glucose uptake is largely insulin‐independent (Illsley & Baumann, 2020). Further, our results do not support a decrease in umbilical glucose supply, uptake by the fetus, or decrease in fetal glucose concentrations. Rather, our results support that the carbon source for increased lactate production in the placenta may be derived from pyruvate produced by the fetus and delivered to the placenta, rather than from pyruvate from glycolysis (glucose) in the placenta. This is further supported by examination of uteroplacental metabolic quotients whereby the glucose quotient is lower, and the pyruvate quotient is higher in HOX fetuses (Table 4). These in vivo uteroplacental nutrient flux measurements are supported by placental tissue data demonstrating decreased expression of glycolytic genes (*PFK1*, *PKM2*), evidence for decreased pyruvate oxidation TCA cycle activity (*MPC2*, *SDHB*), and increased lactate production based on increased LDHA protein expression, increased LDH activity, and decreased *LDHB* gene expression which would favor an LDH enzyme tetramer with more LDHA subunits and promote the conversion of pyruvate to lactate in the HOX placenta. Increased uteroplacental pyruvate uptake from the fetus and increased placental LDH activity in the direction of producing lactate would oxidize NADH and regenerate NAD+ (Rabinowitz & Enerback, 2020) which is necessary to sustain high glycolytic flux in the placenta. Indeed, uteroplacental tissues have a large reciprocal glucose (positive) versus lactate (negative) metabolic quotients (see Table 4, Figure 6), supporting a high‐rate of glucose utilization and lactate production, with a potential greater contribution from pyruvate during hypoxemia. HOX fetuses also had increased arterial lactate and pyruvate concentrations, yet there were no differences in lactate and pyruvate in the maternal circulation, supporting that increased levels of these substrates are a result of fetal or uteroplacental, rather than maternal, metabolism (Boyle et al., 1992; Mann, 1970). HOX fetuses have increased cortisol and norepinephrine concentrations (Jones, Rozance, et al., 2019) which may mediate effects on these fetal‐placental metabolism as other studies in sheep models have shown that experimentally increased maternal cortisol concentrations increase uteroplacental lactate production (Vaughan et al., 2016) and fetal norepinephrine, and decreases in fetal glucose uptake (Davis et al., 2020). Taken together, these results extend earlier speculations about lactate and pyruvate flux during sustained hypoxemia between the placenta and fetus (Mann, 1970) and provide new mechanistic details about the pathways involved in the placenta.

#### 4.1.2 Amino acid metabolism

The flux of alanine from the uteroplacental to the fetus includes alanine from the mother and placental synthesis (Timmerman et al., 1998). Herein, we observed similar uteroplacental to fetal alanine flux rates in CON and HOX groups, supporting that uteroplacental alanine supply for the fetus is not limiting and does not explain decreased fetal alanine uptake. Thus, increased fetal arterial alanine concentrations may result from either increased alanine synthesis or decreased alanine utilization by the HOX fetus. The first may reflect increased de novo fetal alanine synthesis from pyruvate and release from skeletal muscle as suggested during acute 1hr hypoxia in fetal sheep (Walker et al., 2000). The second may reflect increased fetal utilization of lactate, rather than alanine, as a fuel source. In addition, high alanine concentrations can inhibit pyruvate kinase, a rate‐limiting enzyme in glycolysis. Thus, increased alanine in the umbilical circulation of the HOX fetus may inhibit glycolysis in the placenta, consistent with decreased *PKM2* expression and lower expression of other genes in glycolysis (*PFK1*) in the HOX placenta. This effect of alanine may antagonize the putative actions of hypoxemia on increasing glucose utilization in the placenta.

Under normal conditions, the fetus has a net uptake of glycine and glutamine and a net release of serine and glutamate, respectively, which results from exchange between the fetal liver and placenta for these amino acids (Battaglia, 2000; Cetin et al., 1992; Moores et al., 1994; Vaughn et al., 1995). Specifically, glycine is produced by the placenta and released to the fetus, while serine is released by the fetus and taken up by the placenta (Cetin, 2001; Cetin et al., 1991, 1992). Serine concentrations and net serine output were decreased in the HOX fetus. Given the maintenance of glycine uptake by the HOX fetus from the placenta, the decrease in serine output is not limited by glycine supply. Rather, the decrease in fetal serine output may reflect decreased glycine to serine metabolism in the HOX fetal liver or decreased de novo synthesis of serine from glucose. It will be important to understand these effects of hypoxemia on serine‐to‐glycine flux because this exchange is critical for the metabolism and transfer of intermediates in one carbon metabolism between the fetal liver and placenta (Cetin, 2001; Kalhan, 2016). Glutamate uptake by the placenta from the fetus was decreased with hypoxemia, likely as a result of decreased fetal glutamate concentrations and decreased fetal output to the utero‐placenta. This also may result from decreased utilization and oxidation of glutamate in the HOX placenta. Ongoing studies are underway to evaluate the effect of hypoxemia on glutamate‐glutamine, serine‐glycine, lactate‐pyruvate metabolism between the HOX placenta and fetus, and specifically the fetal liver, as it normally releases pyruvate, serine, and glutamate to the placenta, in exchange for lactate, glycine, and glutamine.

#### 4.1.3 Uterine blood flow and oxygen supply

Decreased uterine and uteroplacental blood flow is a common feature in human pregnancies at high altitude and in other models of hypoxemia. Of note, in human IUGR, both glucose and oxygen uptake rates, but not weight‐specific umbilical blood blow rates, were reduced (Cetin et al., 2020). This is in contrast to data in a sheep model of PI‐IUGR, whereby weight‐specific rates of umbilical blood flow and glucose uptake are reduced, while oxygen rates are reduced only in fetuses with severe growth restriction (Regnault et al., 2007; Thorn et al., 2013). In our model, however, sustained hypoxemia during late gestation in pregnancy did not decrease uterine or umbilical blood flow nor increase AMPK activation. Differences between the results from pregnancy studies in women at high altitude and PI‐IUGR compared with our late gestation sheep hypoxemia model may be explained by the duration of exposure with only ~9 days of hypoxemia during late gestation, after placental development is established, compared to human pregnancies affected by chronic and progressive placental insufficiency that may begin as early as the second trimester. Indeed, studies in mice have reported that placental allocation of nutrients is dependent on the timing, duration, and severity of hypoxemia exposure (Higgins et al., 2016). The role of decreased blood flow in driving limited fetal growth remains unclear, especially with recent data demonstrating that, in mice, AMPK activation, but not restoration of blood flow, reduces the magnitude of hypoxia‐associated growth restriction (Lane, Houck, et al., 2020). The maintenance of uteroplacental and fetal oxygen uptake in HOX pregnancies herein, supports that mechanisms are in place to maintain uteroplacental oxygen consumption and that *p*O_2_ or blood oxygen content, rather than oxygen consumption, mediates the effects of hypoxemia in the HOX placenta and fetus. We acknowledge that in our HOX model sheep fetuses were only exposed to hypoxia late in gestation, which may not replicate human IUGR in which hypoxia is of longer duration and produces placental growth impairment and potentially altered mitochondrial numbers or function that limits placental oxygen consumption (Cetin et al., 2020). Further, in the absence of differences in blood flow, our model provides new insight into the flow‐independent changes on uteroplacental nutrient allocation to the fetus. While mTOR signaling in the placenta is important for coordinating the metabolism of amino acids and other substrates (Gupta & Jansson, 2019), we found no differences in the phosphorylation of mTOR or its target proteins, S6 and 4E‐BP1, yet protein levels of 4E‐BP1 were decreased. Additional studies are needed to understand the role of decreased AMPK activation and if decreased 4E‐BP1 expression has an effect on limiting protein synthesis in the HOX placenta.

### 4.2 Summary and implications

Our results support the importance of nutrient shuttles between uteroplacental and fetal tissues which provide metabolic advantages for both tissues to maintain oxidative metabolism in the presence of altered proportions of substrates (Jones, Rozance, et al., 2019). These changes may represent early uteroplacental responses in nutrient allocation to the fetus that allow the fetus to defend its rate of oxidative metabolism and growth. These results further highlight the vulnerability of nutrient sensing and metabolic pathways in the placenta to hypoxemia in late gestation which may have consequences on reducing fetal growth during periods of longer hypoxemia exposures and also may contribute to the developmental programming and increased risk for metabolic disease in offspring exposed to hypoxemia during gestation (Ducsay et al., 2018).

## CONFLICT OF INTEREST

The authors of this manuscript have no conflict of interest to declare.

## AUTHOR CONTRIBUTIONS

All persons designated as authors quality for authorship and are listed. Author contributions to this work include conception or design (SRW and AKJ), acquisition, analysis, or interpretation of data (PJR, LDB, RAL, CGJ, LGM, and SWL), drafting the work (SRW and AKJ), or critically revising intellectual content (PJR, LDB, RAL, CGJ, LGM, and SWL). All authors approved the final manuscript version, agree to be accountable for all aspects of the work to ensure that questions related to the accuracy or integrity of any part of the work is preserved.
